# Genes causative of primary immunodeficiency are risk factors for and are over-expressed in systemic lupus erythematosus

**DOI:** 10.3389/fimmu.2026.1494343

**Published:** 2026-03-03

**Authors:** Haley Davis, Katherine A. Owen, Adam C. Labonte, Erika L. Hubbard, Sophia Kerns, Jessica Kain, Brian Kegerreis, Prathyusha Bachali, Amrie C. Grammer, Peter E. Lipsky

**Affiliations:** AMPEL BioSolutions, LLC, Charlottesville, VA, United States

**Keywords:** autoimmune disease, biomarkers, genetics, lupus, machine learning, primary immunodeficiency disease, systemic lupus erythematosus

## Abstract

**Background:**

Systemic lupus erythematosus (SLE) is a polygenic autoimmune disease characterized by the production of autoantibodies leading to widespread inflammation, whereas primary immunodeficiency (PID) disease is a group of specific genetic defects in molecular pathways required for host defense to infectious agents. PID genes are found to play critical roles in development and function of the immune system, and decreased function results in dramatically increased susceptibility to infection. We hypothesized that PID genes would be genetically over-represented and over-expressed in SLE, facilitating the identification of novel risk genes and molecular pathways involved in lupus pathogenesis.

**Methods:**

A comprehensive database of 453 PID genes was developed. PID genes were clustered into protein-protein interaction (PPI) networks and compared to known SLE risk loci. Over-expression of PID genes was examined in datasets from multiple sample types. Machine learning (ML) classifiers using PID genes as input were employed to predict SLE disease status and severity.

**Results:**

PPI clustering of PID genes revealed 18 distinct cellular and functional groups. PID genes overlapped with SLE risk loci more than expected by random chance and in greater magnitude than PID gene overlap with risk alleles of other autoimmune conditions. In both whole blood and immune cell-specific samples, PID genes were significantly differentially expressed (DE) more than expected by random chance, with most of these genes being over-expressed in SLE. Specific patterns of DE genes, according to the cellular and functional groups, were mapped to expression by specific immune cell subsets, with many PID genes upregulated in T cells, classical monocytes, and non-classical monocytes. Over-expression of and enrichment of PID genes and functional clusters were also positively associated with increased measures of lupus disease activity. Finally, functionally different groupings of PID genes were useful in classifying lupus from healthy controls and active lupus from inactive lupus by ML with accuracies of 0.80 and 0.74, respectively.

**Conclusions:**

PID genes are not only genetically associated with SLE but are also broadly over-expressed, particularly in active disease. These results highlight the profound link between pathways governing host defense and SLE immunopathogenesis, and PID genes may be considered further for therapeutic targeting.

## Introduction

SLE is a chronic, female-biased autoimmune disease defined by the production of high affinity autoantibodies that cause inflammation and dysfunction in many organs, including the skin, kidneys, lungs, central nervous system and hematopoietic system. There is extensive evidence that genetics plays a role in both SLE susceptibility and severity, but the genetic contribution to SLE is complex. Genome-wide association studies (GWAS) have identified more than 100 risk loci with a *p* value <5 × 10^−8^, and these loci identify mechanisms and pathways that may contribute to and/or coincide with disease pathogenesis (1–6). However, these loci are thought to represent less than 30% of disease heritability (7).

Although SLE is commonly thought of as a polygenic disease with modulatory epigenetic features, monogenic lupus has also been reported in a small subset of patients, usually presenting at a very young age (less than 5 years). More than 30 single gene variants have been identified to cause monogenic lupus or a lupus-like phenotype, and these have been important in defining potential pathogenic mechanisms that might contribute to polygenic SLE. Genes underlying monogenic lupus include *TREX1*, *SAMHD1*, *RAG2*, *FAS*, *FASL, IRF7* and various complement components (C1q, C2, C4) (8). Additional rare variants might also contribute to monogenic lupus (9).

An alternative way to identify the genetic basis of a disease, such as SLE, is the candidate gene approach, in which a specific molecular pathway likely to be associated with the disease is identified from animal models or literature mining and the genes of non-redundant regulators tested for the relationship to disease manifestations. Results from the candidate gene approach have been helpful in identifying specific, SLE-related genes (10, 11). This approach has been expanded to include networks of genes, such as the so-called immunome (12). Here, we have expanded this approach to examine a large series of candidate genes, namely those that have been shown to be causative in human primary immunodeficiency (PID) disease. These genes have been found to play critical roles in development and function of the innate and adaptive immune system, and decreased function of these genes results in dramatically increased susceptibility to infection (13, 14). Since the basis of SLE resides in hyperactive immune function, we hypothesized that PID genes would be over-represented in SLE risk variants and also over-expressed in SLE and that varying expression patterns may implicate specific immune pathways involved in lupus pathogenesis.

PID is a series of diseases linked by the genetic predisposition for increased susceptibility to infection with one or more classes of causative infectious organisms. Specific PID diseases are characterized by developmental defects or functional inactivation of the adaptive and/or innate immune system, in which the causal genes encode nonredundant steps in controlling infection (15). PIDs are rare, with an incidence of approximately 1 in 1200 births in the United States, but the genes involved clearly indicate a necessary step in human host defense (16). It is noteworthy that a link between PID and autoimmunity has been suggested, since one or more autoimmune or inflammatory conditions was observed in approximately 25% of patients with PID over their lifetime (17). PID diseases with coupled autoimmune disorders include B-cell immunodeficiency (XLA, CVID, and Selective IgA deficiency), common immunodeficiency (Wiskott-Aldrich syndrome) and deficiency in early and late complement pathway components. A link between PID and SLE has also been suggested, since complement deficiencies (C1q, C1r, C2, C4, C5, C6, C7, C8, and C9 mutations) frequently present with autoimmune disease and especially SLE (16). In addition, CGD (chronic granulomatous diseases), idiopathic CD4+ lymphocytopenia, and ALPS (autoimmune lymphoproliferative syndrome) present with some clinical manifestations of SLE (18).

In the current study, we compiled a database of 453 genes found to be causative of PID. Notably, a subset of these genes, including *STAT4*, *IRF7*, *ITGAM*, *IRAK4* and *TYK2*, were predicted to be lupus susceptibility genes from GWAS (4, 5). Here, we found that 28.5% of the PID genes are risk genes for SLE. PID genes also overlap with risk variants associated with rheumatoid arthritis and multiple sclerosis, but to a lesser extent. Networks of genes predicted from protein-protein interaction mapping indicated that PID genes largely clustered into categories defining adaptive and innate immune functions. Of the 453 PID genes, as many as 335 (74%) were differentially expressed in lupus samples and of the differentially expressed genes, 280 (61.8%) were over-expressed. PID genes were predominantly over-expressed in patients with active lupus. Using PID-defined gene modules as features, machine learning (ML) models could successfully classify SLE from healthy controls and active from inactive SLE, although the most important features differed in each classification. The data suggest that many PID genes are risk factors for SLE and that most PID genes are over-expressed in SLE, highlighting the common but opposing nature of the immune and inflammatory pathways essential for host defense on the one hand and immunopathogenesis of SLE on the other.

## Materials and methods

### Construction of the PID gene database

Monogenic causal PID genes were identified by a thorough search of primary scientific literature, including published studies in PID genetics, PID gene databases, regular reports from PID gene classification panels and gene mutation phenotype databases. Once identified through this mining technique, genes were organized into a database that included the following information for each gene: Gene Symbol, Official Symbol, Full Name, Functional Category (BIG-C [Biologically Informed Gene Clustering], see below), Entrez ID, Ensembl ID, Gene Type, Synonyms, Chromosome Number, Cytogenetic Location, Inheritance, genetic Defect/Pathogenesis, Phenotype, Relevance to SLE, Allelic Mutations (OMIM and Primary literature), Protein Effect (GeneCards), OMIM Gene ID, OMIM Phenotype ID and Mendelian Genetics ID (**Supplementary Table S1**).

### Compilation and analysis of SLE patient gene expression data

Data were derived from publicly available datasets and collaborators (**Supplementary Table S2**). Raw data files were obtained from the GEO repository for SLE whole blood data. Whole blood-derived datasets GSE45291 and GSE88884 were selected based on the criteria that both SLE patients and healthy controls are included and that both are relatively large in terms of patient number. GSE49454 was also included for additional confirmation and supplementary analyses. In addition, these datasets contain both active (SLEDAI ≥ 6) and inactive (SLEDAI < 6) SLE patients.

GSE45291 includes 266 female patients (34 active and 232 inactive) and 20 controls. Data for GSE45291 were collected at baseline and include various ancestral backgrounds (Asian, African American, European American, others). Data processing and differential expression (DE) analysis was conducted using the LIMMA package within the R Suite. Affymetrix CEL files underwent background correction and GCRMA normalization based on annotations using either the onboard Affymetrix chip definition file (CDF) or the hgU133plus2 Entrez Brainarray CDF. Outliers were identified through inspection of the first, second, and third principal components used as axes in a three-dimensional PCA plot, and through inspection of array dendrograms calculated using Euclidean distances and clustered using average/UPGMA agglomeration (unweighted pair group method with arithmetic mean). The LIMMA package was utilized to create linear models of gene expression through empirical Bayesian fitting. The Affymetrix CDF and Brainarray CDF expression sets were analyzed separately. For each, a design matrix was created based on disease status, linear models fitted, and the SLE/normal contrast (expression ratios) extracted for analyses. DE analysis was carried out using moderated t-statistics with related p-values adjusted using Benjamini-Hochberg multiple hypothesis testing. Probes with duplicate gene symbols were removed by retaining the probe with the lowest unadjusted or adjusted p-value, depending on which p-values yielded statistical significance (Affymetrix CDF or Brainarray CDF significant genes were identified using an unadjusted p-value < = 0.05 or an adjusted p-value < 0.2). The two significant CDF lists were merged and duplicate probes were removed by retaining the most significant probe.

We analyzed female patients from GSE88884, including 1620 individuals with SLE of various ancestral backgrounds (African American, American Indian, Asian, Pacific Islander, Caucasian, and Mixed) split into two groups, Illuminate 1 and Illuminate 2, derived from study NCT01196091 and study NCT01205438, respectively. Data processing and analysis were conducted similarly for GSE45291.

### RNA-seq data

Raw data files were downloaded from the NCBI Sequence Read Archive (SRA) website using the SRA toolkit (version 2.10) and converted to FASTQ files using fastq dump. Quality of the FASTQ files was checked using FASTQC software (version 0.11.9). Adapters and poor-quality reads were trimmed using Trimmomatic software (Unix based tool version 0.38). Good quality reads were aligned to the human reference genome (hg38) using the STAR aligner (version 2.7). STAR-aligned reads were saved as sam files and were converted to bam files using sambamba (version 0.8). Read counts were summarized using featureCounts function of the Subread package (version 1.61). Count normalization and log transformation were carried out using DESeq2 (version 1.32) R package.

### Functional annotation of genes via IScope and TScope

IScope is a cellular aggregating tool that categorizes gene transcripts into 32 possible hematopoietic cell categories based on matching 926 transcripts uniquely expressed in hematopoietic cells and known to mark various types of immune/inflammatory cells (19). TScope is an additional aggregation tool to characterize cell types found in specific tissues in which transcripts are sorted into one of 8 categories representing a specific tissue or tissue cell subtype based on matching 704 total transcripts. Genes in the PID database were cross-referenced with the IScope and TScope categories for immune cell and tissue cell types.

### Visualization of chromosomal location of PID genes and SNP-implicated SLE risk genes with Circos

The chromosomal location of PID genes and single nucleotide polymorphism (SNP)-implicated SLE risk genes (SISRGs) along with their overlap were visualized via Circos. Circos diagrams were generated using Circa Genomics Software version 1.2.2. The human hg38 chromosome assembly (GRCh38) was used as a scaffold and gene base pair coordinates were obtained from the BioMart repository for GRCh38.

### Cross reference to GWAS-defined genes

Monogenic causal PID genes were cross-referenced against SLE susceptibility genes independentlyderived from the SLE Immunochip GWAS as described (4, 5). In brief, SNPs in high association with SLE incidence were matched to proxy genes via linkage disequilibrium in order to identify corresponding expression quantitative trait loci (eQTLs) using GTEx v.6 and mapped to their associated expression genes (E-genes). SLE-associated SNPs occurring in transcription factors or downstream target genes (T-genes), protein-coding genes (C-genes), or proximal genes (P-genes) were identified via appropriate gene databases (Hacer, GeneHancer, Ensembl genome browser, dbSNP). A total of 5,489 SISRGs were identified for cross reference against PID genes. Frequency of PID genes identified in this overlap was compared to frequency of overlap with randomly selected sets of genes (451 for all genes on the chip or 427 when limited to protein coding genes) via Monte Carlo simulation with 10,000 iterations as described below. A similar pipeline was employed to identify causal genes for rheumatoid arthritis (RA) and multiple sclerosis (MS) using GWAS results from GCST002318 and GCST005531, respectively, and used to overlap with PID genes. A total of 822 MS SNP-predicted genes and 717 RA SNP-predicted genes were identified and used for comparison with PID genes (**Supplementary Table S3**).

### Monte Carlo simulations

Monte Carlo simulations were carried out to determine the expected number of PID genes in gene sets of specific sizes, in efforts to determine whether the 129, 36, and 37 PID genes within the 3,106, 944, and 1,029 genes predicted from SNPs associated with SLE, RA (GCST002318), and MS (GCST005531), respectively, were more than expected at random. Random genes were selected from 19,438 unique HGNC symbols corresponding to protein-coding genes on biomaRt. Simulations were based on the numbers of SISRGs and PID genes included in the 19,438 HGNC symbols: 129 PID genes in 2,082 SISRGs, 35 PID genes in 586 RA SNP-predicted genes, and 36 PID genes in 605 MS SNP-predicted genes. For each number of SNP-predicted genes (2,082, 586, and 605), 10,000 random gene sets were generated and the number of PID genes in each set was recorded. With 10,000 simulations for each gene set size, the mean and standard deviation was calculated and used to determine the z-score (i.e., z-score = [true overlap (128, 35, and 36) - mean random overlap]/standard deviation of random overlap).

### Protein-protein interaction network construction and cluster creation

Visualization of protein-protein interactions and relationships between genes within datasets was done using Cytoscape (V3.6.0) software and the mCODE StringApp (V1.3.2) plugin application. The Clustermaker2 App (V1.2.1) plugin was used to create clusters of the most related genes within a dataset using a network scoring degree cutoff of 2 and setting a node score cut-off of 0.2, k-Core of 2 and a max depth of 100. DE cell type comparison plots were generated by importing DE values (i.e., log-transformed expression values) from six datasets (whole blood [WB], GSE39088; peripheral blood mononuclear cells [PBMC], GSE50772; CD14^+^CD16^-^ classical monocytes, GSE51997; CD14^+^CD16^+^ nonclassical monocytes, GSE51997; CD19^+^ B cells, GSE4588; and CD4^+^ T cells, GSE51997) as individual node attribute columns and assigning node color to these values with continuous mapping.

### Enrichment of functional groups of genes in mCODE-generated clusters

Enrichment of groups of genes in mCODE-generated clusters were calculated with Fisher’s Exact Test and Biologically Informed Gene Clustering (BIG-C) categories. BIG-C is a functional aggregating tool that sorts genes into one of 52 categories based on their canonical biological function and/or cellular localization by utilizing information from multiple online tools and databases (20, 21). Bubble Plots were generated using a custom typescript application that simultaneously graphs enrichment odds ratios (circle size) and -log(p) values (circle color).

### Gene set variation analysis

The GSVA (V1.25.0) software package for R/Bioconductor was used as a non-parametric, unsupervised method for estimating the variation of pre-defined gene sets in patient and control samples of microarray expression datasets. GSVA was run using GSE88884 and the mCODE clusters. Hedge’s g values, a measure of effect size, were calculated from GSVA enrichment scores by contrasting scores of all controls against all lupus patient samples. GSVA enrichment scores were additionally analyzed with Welch’s t-test to identify significant (p < 0.05) gene categories contributing to substantial segregation of cohort samples. Results were visualized by using a matrix of Hedge’s g values entered as input to a dual scale heatmap2 function in R. Significant categories are denoted by asterisks.

### Evaluation of patient groups by DE-based and GSVA-based hierarchical clustering

Expression values of DE PID genes or GSVA enrichment scores of mCODE-derived PID gene clusters for each patient within Illuminate-1 or Illuminate-2 were subjected to hierarchical clustering calculated using Euclidean distances and complete linkage. After inspection, the clustering was repeated with k=3 to establish three patient clusters. For each cluster, mean and standard deviation of each of the following clinical traits were calculated: SLE Disease Activity Index (SLEDAI), anti-dsDNA titers (International units [IU]), serum C3 levels (g/L), and serum C4 levels (g/L). Statistical significance of results from each group was calculated with one-way ANOVA followed by Tukey’s test.

### Generation of Illuminate patient groups by Gaussian mixture variational autoencoder

Partitioning around medoids was used to derive phenotypic-based clusters from the combined patient pool of Illuminate-1 and Illuminate-2 based on clinical metadata (SLEDAI, age, alopecia, anti-dsDNA, low complement, ulcers, antimalarial treatment, corticosteroid treatment, immunosuppressant treatment, NSAID treatment, active drug and placebo treatment). A Gaussian mixture variational autoencoder was then trained on clinical data from GSE88884 to identify five classes (number of classes chosen by examination of a Bayesian information criterion plot).

### Feature selection and machine learning

For the feature selection analysis, the normalized log2 gene expression matrix (e-set) of each dataset and the PID gene sets (i.e., PID gene mCODE clusters) were used as the input. GSVA was run on each dataset separately. Low intensity genes were filtered and only those with IQR > 0 across all the samples were considered in the analysis. GSVA was carried out separately for lupus samples vs. healthy controls and active lupus samples (SLEDAI ≥ 6) vs. inactive lupus samples (SLEDAI < 6). GSVA enrichment scores, that range from -1 to 1 from every dataset were concatenated from multiple datasets (GSE88884 Illuminate-1, GSE88884 Illuminate-2, GSE45291, GSE39088, and GSE112087), providing a sufficiently large cohort for feature extraction and to stratify lupus patients based on disease activity.

Various feature selection techniques were employed to remove the noise and select features which contribute most to the prediction variable. The concatenated GSVA score matrix was used as input. The analysis was carried out as follows: Two GSVA matrices were concatenated and designated as 1) Discovery Cohort 1–1936 lupus samples 96 normal donors (GSE88884 Illuminate-1, GSE88884 Illuminate-2, GSE45291, GSE39088, GSE112087); and 2) Discovery Cohort 2–1665 active lupus samples 242 inactive lupus samples (GSE88884 ILL-1, GSE88884 ILL-2, GSE45291, GSE39088, GSE112087). Feature extraction was carried out in Python using scikit-learn (V 0.24.1) independently on Discovery Cohort 1 and Discovery Cohort 2 and involved removing missing features and any features with low variance across all samples of each cohort.

Two independent binary classifications of Discovery Cohort 1 and Discovery Cohort 2 were carried out using scikit-learn in Python (V 3.8.2). Several linear, nonlinear, and ensemble ML algorithms such as Logistic Regression (LR), K-Nearest Neighbor (KNN), Naïve Bayes (NB), Support Vector Machine (SVM), Random Forest (RF), Gradient Boosting (GB), Decision Tree (DTREE), Linear Discriminant Analysis (LDA), and Adaptive Boosting (ADB) were implemented to distinguish lupus from normal donors and active lupus samples from inactive lupus samples. The performance of various binary classifiers was evaluated based on sensitivity, specificity, Cohen’s kappa, f1-score, and accuracy. Because of imbalances in the number of SLE and normal samples in the cohorts, sub-sampling without replacement was employed by creating 20 different folds by random selection of 96 SLE samples to match with the minority class (controls) in Discovery Cohort 1 and by creating 7 folds by randomly selecting 242 active lupus samples to match with the minority class (inactive lupus) in Discovery Cohort 2. The data from each fold were split into 70% training and 30% validation. Various ML classifiers were built on training data and evaluated on validation data. One-vs.-one and one-vs.-rest multi-class classifications with leave-one-out cross-validation were employed to predict class (lupus or active lupus). Average performance measures, including, sensitivity, specificity, accuracy, f1-score and Cohen’s kappa, were calculated from all 20 folds of Discovery Cohort 1 and 7 folds of Discovery Cohort 2. Receiver Operating Characteristic (ROC) curves and Precision-Recall (PR) curves were plotted using the matplotlib (V 3.3.4) library in Python. The permutation importance function from SVM was used to calculate feature importance scores to identify the top predictors of each classification.

## Results

### Characterization of primary immunodeficiency genes

**Figure 1:**
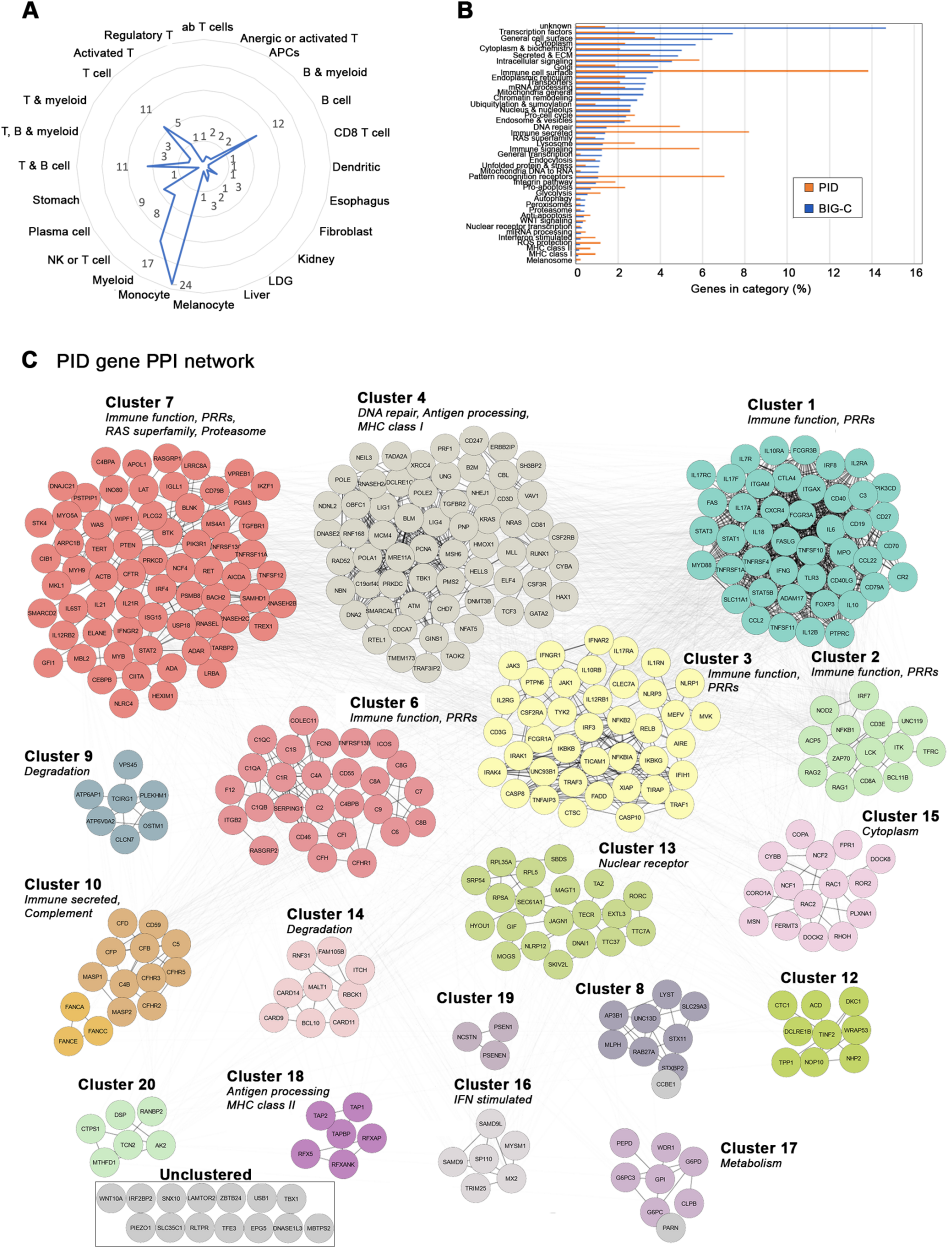
Biological characterization of the primary immunodeficiency gene database. Breakdown of primary immunodeficiency database contents by cell type/tissue of origin and biological function. **(A)** Tissue and cell type enrichment shown as gene count for each IScope/TScope category. **(B)** Biological functions represented within the database are displayed as percentage of total PID genes present in each BIG-C category (orange). Native breakdown of all genes represented within the BIG-C tool is shown as percentage of total BIG-C genes present in each functional category (blue). **(C)** Interaction network of genes present within the PID gene database. Genes are colored according to mCODE cluster membership. Major cluster functional annotations are listed.

A total of 453 PID genes were compiled into a database and used for the current analysis (**Supplementary Table S1**). To establish the likely cells affected by PID genes, we aggregated all 453 genes and assessed them using IScope and TScope to determine the cells most likely to express them (22). Cell types predicted from the aggregated PID gene database were biased toward immune effector cell types with 125 genes specific for 25 hematopoietic immune cell categories, in particular, monocytes, myeloid cells, B cell, and T cell lineages (**Figure 1A**). Far fewer genes were assigned to tissue-specific categories, with only 1 kidney gene, 3 liver genes, and 1 skin (melanocyte)-specific gene. The remaining genes, although not cell-specific enough to signify distinct cell lineages, were largely identified as members of immune-related functional categories, for example, pattern recognition receptors (PRRs), interferon stimulated genes, and secreted immune categories (**Supplementary Table S4**). Analysis of clinical phenotypes associated with each PID gene in the database revealed that, of PID genes whose effects could be traced to specific immune cell populations, 64.71% matched the population implicated in this molecular analysis. When limited to only PIDs with identified cellular phenotypes specifically affecting B, T, and plasma cell populations, an 80% overlap with the molecular-based results was observed (data not shown). The most likely functions of the PID genes were also analyzed by assessing their membership in biologic pathways using functional annotation provided by the BIG-C tool (**Supplementary Figure S1**). Importantly, the enrichment of PID genes in these specific categories was distinctly different than the categorical distribution of all BIG-C genes themselves, indicating a marked skewing of PID genes toward immune functions (**Figure 1B**).

To identify patterns and potential signaling pathways represented by PID genes in greater detail, protein-protein interaction (PPI) networks were generated. The 453 PID genes grouped into 20 mCODE-derived clusters with varying sizes and degrees of interconnectivity (**Figure 1C**). Two of the clusters contained only one gene, and, therefore, were not further considered, leaving 18 final PPI PID clusters that were assessed. Functional gene category enrichment (**Supplementary Figure S1**; **Figure 1C**) indicated that five of the largest clusters (1, 2, 3, 6, and 7) were all dominated by functional molecular categories common to immune cell lineage signatures, including immune cell surface, immune signaling, secreted immune and PRRs. In addition to immune function, large cluster 7 was enriched in a number of categories representative of general cell function (RAS superfamily, proteasome, cytoskeleton, endocytosis and golgi) as well as processes related to antigen processing (MHC class II) and degradation (proteasome, unfolded protein and stress). Strikingly, antigen processing was also characteristic of clusters 4 (MHC class I) and 18 (MHC class I and II), highlighting the importance of these pathways for normal immune function. Other notable immune-related clusters included cluster 16, that was strongly enriched specifically for IFN-stimulated genes, and cluster 10, enriched in secreted complement factors (secreted and ECM). The remaining non-immune PID clusters were dominated by a wide range of functions, indicative of metabolism (cluster 17), degradation (clusters 9 and 14) and the cytoskeleton (cluster 15), amongst others. Thus, numerous molecular pathways involved in both immune and general cell functions implicated by the set of PID genes contribute to the diversity of phenotypes observed in PID patients.

### SNP-implicated SLE risk genes overlap with PID genes

**Figure 2:**
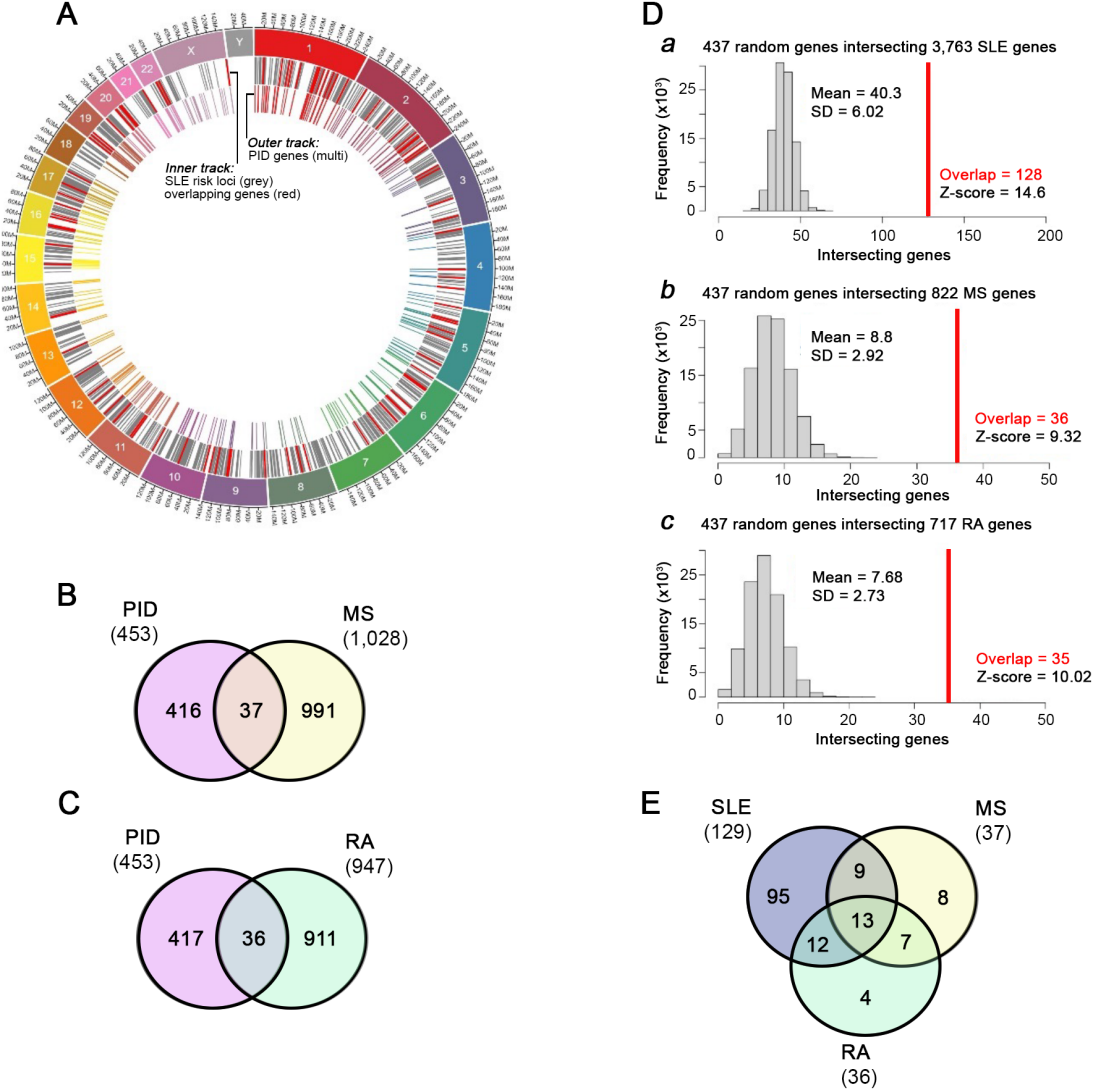
Overlap between PID genes and SNP-predicted SLE risk genes. Circos plot **(A)** showing locations and overlap of SNP-predicted SLE risk genes (grey track) and PID genes (multicolored track). Overlapping genes are highlighted in red. **(B, C)** Overlap of PID genes with SNP-predicted genes from multiple sclerosis **(B)** and rheumatoid arthritis **(C)** GWAS. **(D)** Monte Carlo analysis of the probability of overlap of randomly selected genes with SNP-predicted genes in SLE (128, panel a), MS (36, panel b), and RA (35, panel c). Simulations were performed using only genes contained within the HGNC database as described. **(E)** Venn diagram of all overlapping genes across SLE (129), MS (37) and RA (36). The total numbers in the Venn diagram **(E)** may differ slightly from the text, as the diagram includes all overlapping genes identified, whereas the Monte Carlo simulation was restricted to protein-coding genes available in the HGNC database.

To determine whether PID genes were present among the independently identified causal genes predicted from SLE-associated SNPs, we calculated the overlap between genes in the PID database and a database of 3,763 genes predicted from risk loci identified by multiple large-scale SLE GWAS (**Figure 2A**; **Supplementary Table S5**) (4, 5). Out ofa total of 453 PID genes, 129 (28.5%) were SISRGs, including 9 SLE genes (*CCL22, CR2, GIF, IFIH1, IRAK1, ITGAM, TNFAIP3, TNFRSF13B* and *TYK2*) in which the nucleic acid alteration was located in a coding region (C-Gene) resulting in a nonsynonymous amino acid change, and 36 SLE genes in which the SNP was located in a regulatory region connected to a downstream target (T-) gene (**Supplementary Table S5**). GWAS datasets from two independent autoimmune diseases, multiple sclerosis (MS, GCST005531) and rheumatoid arthritis (RA, GCST002318) were used to determine whether the gene overlap of PID was specific to SLE. Overlap between both MS-associated genes (37, or 8.2%) and RA-associated genes (36, or 7.9%) was significantly greater than expected from random chance (**Figures 2B–D**), but the degree of overlap between PID genes and SISRGs was substantially greater (z-score = 14.6 for SLE, versus 9.3 and 10.0 for MS and RS, respectively) and is likely related to the shared immune activity (**Figure 2E**; **Supplementary Table S5**).

**Figure 3:**
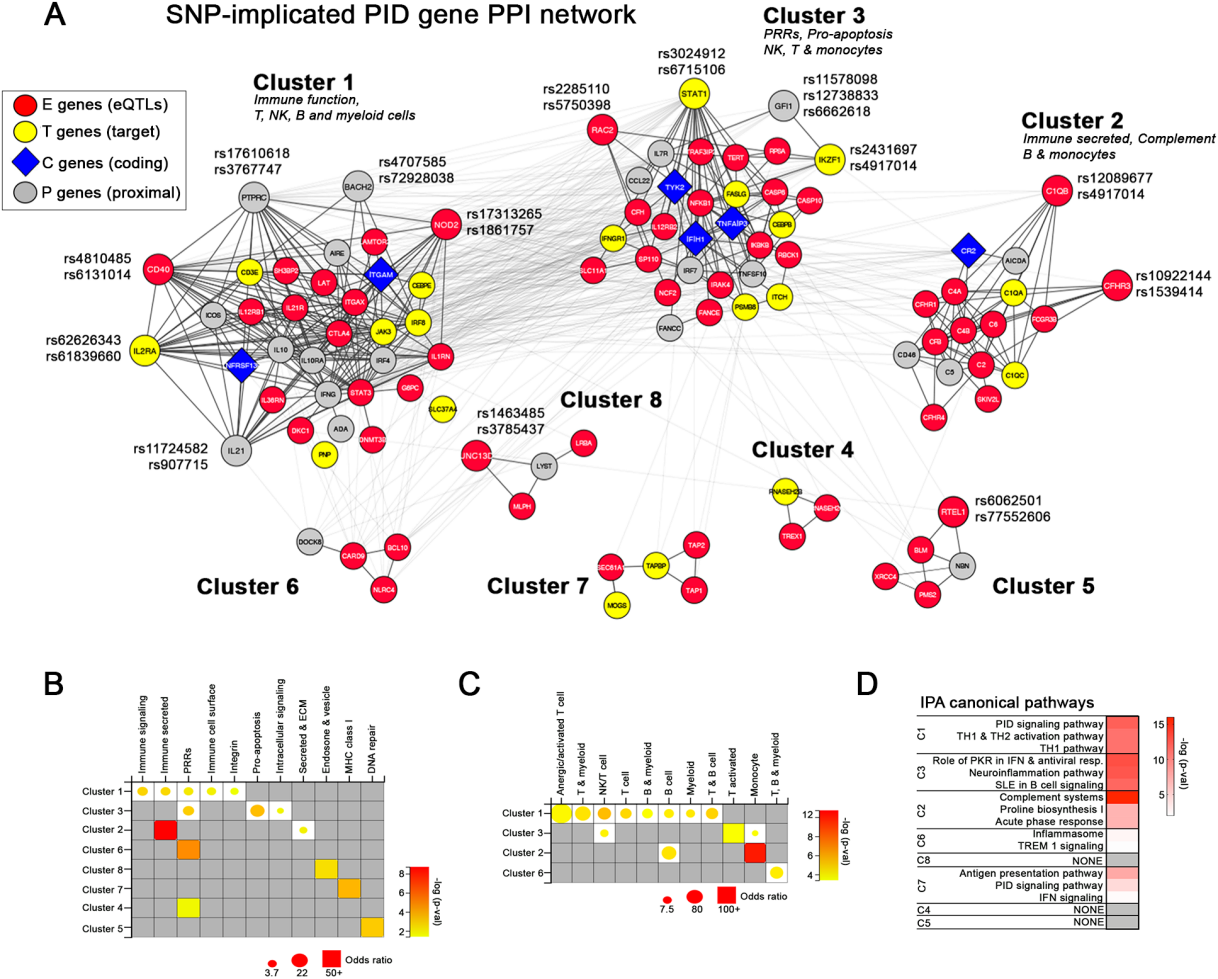
Protein-protein interaction network of SNP-predicted SLE risk genes. Interaction network **(A)** of SNP-implicated SLE risk genes (SISRGs) generated in Cytoscape and clustered via mCODE. Genes are annotated by type (red circles, E-genes; yellow circles, T-genes; blue diamonds, C-genes; grey circles, P-genes) and genes identified directly by SLE risk SNPs are labeled with SNP reference number. **(B, C)** Bubble plots showing cluster enrichment of BIG-C functional categories **(B)** and IScope cell category **(C)**. Odds ratio is shown by bubble size and significance is shown by bubble color shading as -log(p). **(D)** Top pathways for each cluster by IPA canonical pathway analysis.

To learn more about the biological relationships among the subset of PID genes that overlapped with SISRGs, overlapping genes were integrated into a PPI network and grouped into clusters with the mCODE plugin (**Figure 3A**). This method resulted in three larger and five small clusters composed primarily of genes exhibiting expression quantitative trait loci (eQTL) effects (expression (E)-Genes) and genes proximal to a risk SNP (P-Genes). Interestingly, many genes, including *CD40*, *STAT1* and *C1QB* are implicated by multiple SLE SNPs (4). Of the three large clusters, cluster 1 contained a high proportion of surface-expressed immune markers (e.g., CD40, IL2RA, TNFRSF13B, ICOS, IL21R) and secreted immune factors (e.g., IL10, IL21, IFNG) as well as several pattern recognition receptor genes such as NOD2, IRF4, and IRF8; cluster 2 contained multiple complement genes; and cluster 3 contained several key intracellular signaling and IFN response genes, including, IRAK4, IRF7, and IFIH1 (**Figures 3A, B**). Cell type enrichment analysis showed that cluster 1 was broadly enriched for genes from nearly all immune cell types, whereas cluster 2 was very specifically enriched in monocytes and B cells, and cluster 3 was enriched in NK cells and activated T cells (and to a lesser extent monocytes) (**Figure 3C**). Pathway analysis confirmed that the key pathways represented in the SISRGs overlapping with PID genes in clusters 1, 2, and 3 were Th1/Th2 activation pathway, complement systems, and IFN signaling, respectively (**Figure 3D**).

### PID genes are enriched in differential expression datasets from SLE patients

**Figure 4:**
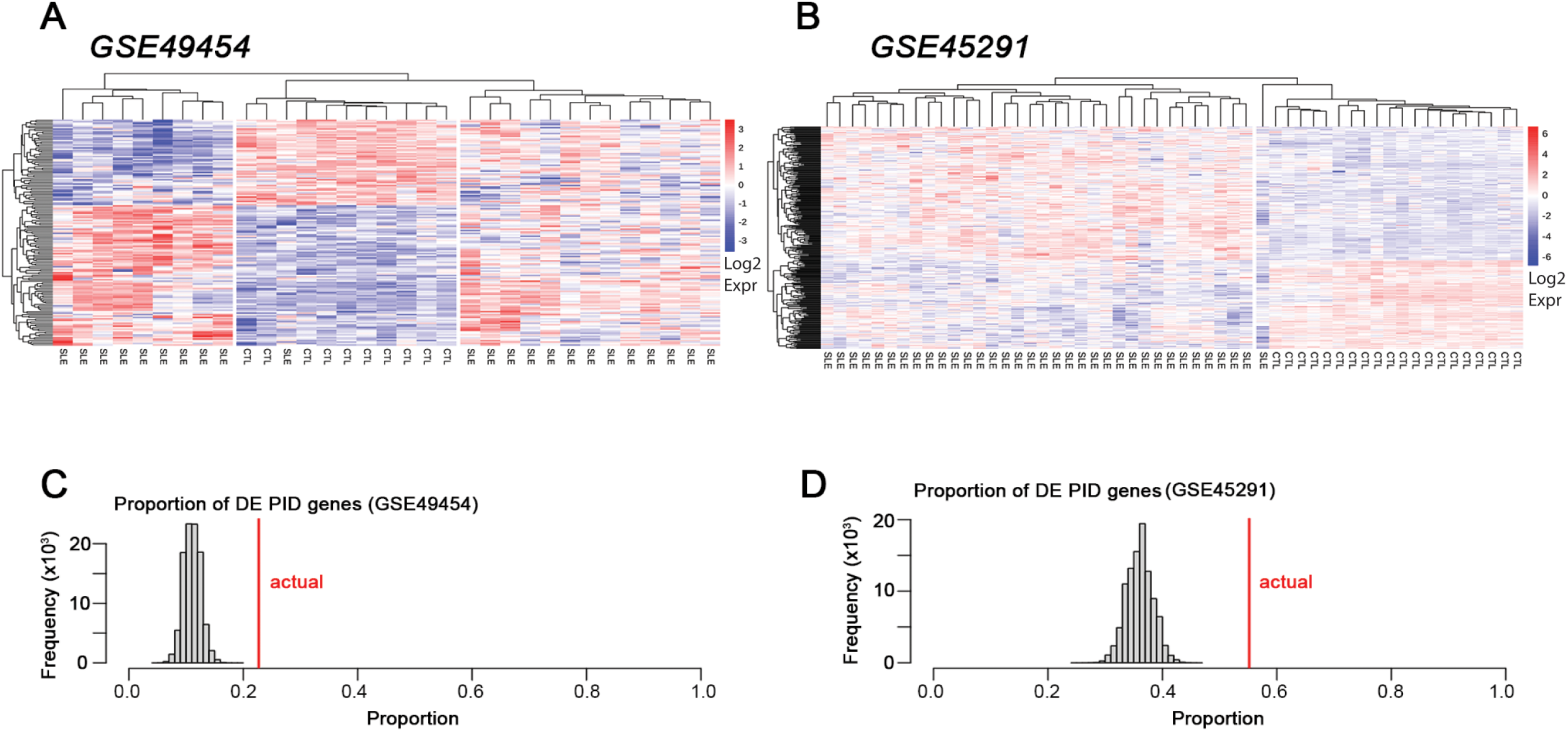
PID genes are significantly differentially expressed in SLE patients. DE PID genes from two lupus datasets, **(A)** GSE49454 and **(B)** GSE45291. Over-expressed genes are shown in red, under-expressed genes are shown in blue. Patient cohort (SLE or healthy control) is indicated at the bottom of each column. Results are shown following directed hierarchical clustering after observing unsupervised hierarchical clustering. **(C, D)** Monte Carlo simulation results for random gene overlap with SLE patient DE genes. Simulations against random samples from the pool of all genes present on microarray were run 100,000 times each and resulting number of overlapping genes are shown as histograms. Red lines indicate actual proportion of DE PID genes for each dataset.

In addition to the striking overlap between PID genes and SISRGs, PID genes were also differentially expressed in lupus patients from two independent whole blood data sets when compared to healthy controls. By hierarchical clustering based on expression of PID genes, SLE samples clearly were separated from normal subjects in both datasets. In GSE49454, SLE patients divided into two groups, with one cluster exhibiting approximately 60% upregulated and 40% downregulated PID genes and the second cluster of SLE patients exhibiting a more varied picture, but both clearly separated from normal (**Figure 4A**).

In GSE45291, only one group of SLE samples was noted, which was clearly distinguished from normal, again with about 60% of PID genes over-expressed (**Figure 4B**). Overall, the majority of PID genes were consistently over-expressed in the SLE cohorts (**Figures 4A, B**) and this was significantly (p < 0.0001) greater than expected from random chance as determined by Monte Carlo simulations. (**Figures 4C, D**).

**Figure 5:**
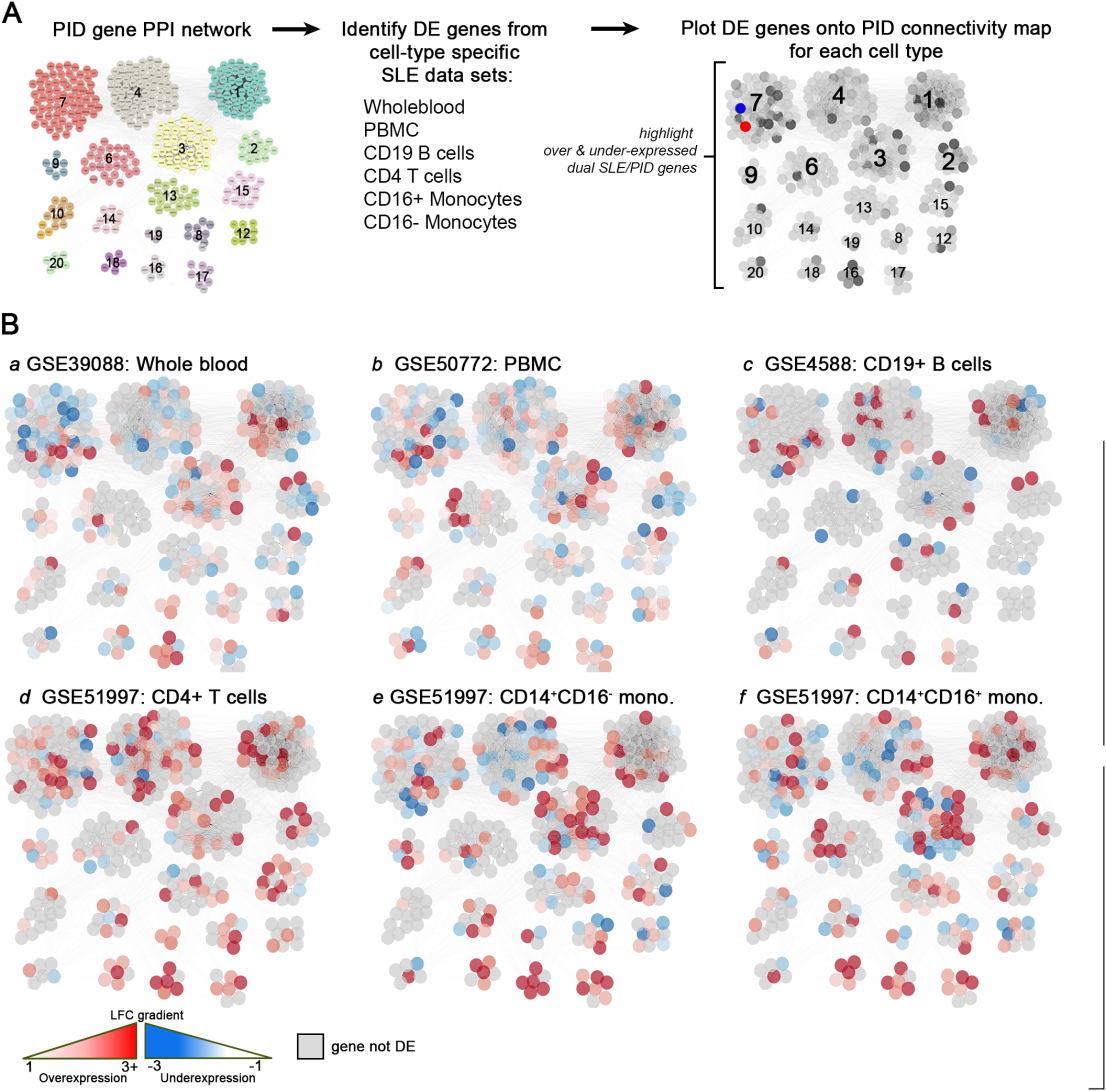
PID mCODE clusters show unique expression patterns among immune cell populations. Summary workflow diagram **(A)** showing the identification of differentially expressed genes from cell-type specific SLE datasets plotted onto the PID gene PPI network template. **(B)** DE data from sorted cell datasets [whole blood, panel **(a)**, PBMC, panel **(b)**, CD19+ B cells, panel **(c)**, CD4+ T cells, panel **(d)**, CD14+CD16- classical monocytes, panel **(e)** and CD14+CD16+ non-classical monocytes, panel **(f)**] overlayed on the PID mCODE network. Each node represents one gene, with over-expressed genes shown in red and under-expressed genes shown in blue. Genes that were not significantly DE are shown in gray.

Given the high percentage of PID genes that were differentially expressed in SLE whole blood, we next sought to determine whether PID genes were differentially expressed in specific cell populations. Expression data from multiple immune cell datasets, including PBMCs, B cells, T cells and monocytes isolated from SLE patients were individually plotted onto the PID gene PPI network (described in **Figure 1C**) to highlight over- and under-expressed genes for each cell type (**Figure 5A**). The expression patterns between the WB and PBMC datasets seemed broadly conserved, including mixed but generally downregulated PID gene expression in cluster 7, moderate gene upregulation in clusters 1 and 3, and moderate upregulation in several small clusters including 8, 9, 10, 16, and 17 (**Figures 5B**, panels a and b). Of the two, PBMC displayed slightly enhanced PID upregulation compared to WB in clusters 3, 6, 9, 10, 17, and 20 (**Figure 5B**, panels a and b). Comparing PID expression patterns of classical (CD14^+^CD16^-^, panel e) and nonclassical (CD14^+^CD16^+^, panel f) monocytes revealed that both subpopulations strongly upregulate PID genes across almost all clusters, and nearly identical cluster-based expression patterns suggest that their expression of PID genes are highly similar (**Figure 5B**, panel d). T cells also exhibited a highly positive PID gene expression profile across all clusters (**Figure 5B**). Interestingly, cluster 3 was most highly enriched in myeloid cell-derived DE genes, whereas cluster 4 was enriched in genes from lymphoid cells (**Figures 5B**, panels c through f).

### PID gene signatures are differentially enriched in lupus patients and can be used to predict disease severity

**Figure 6:**
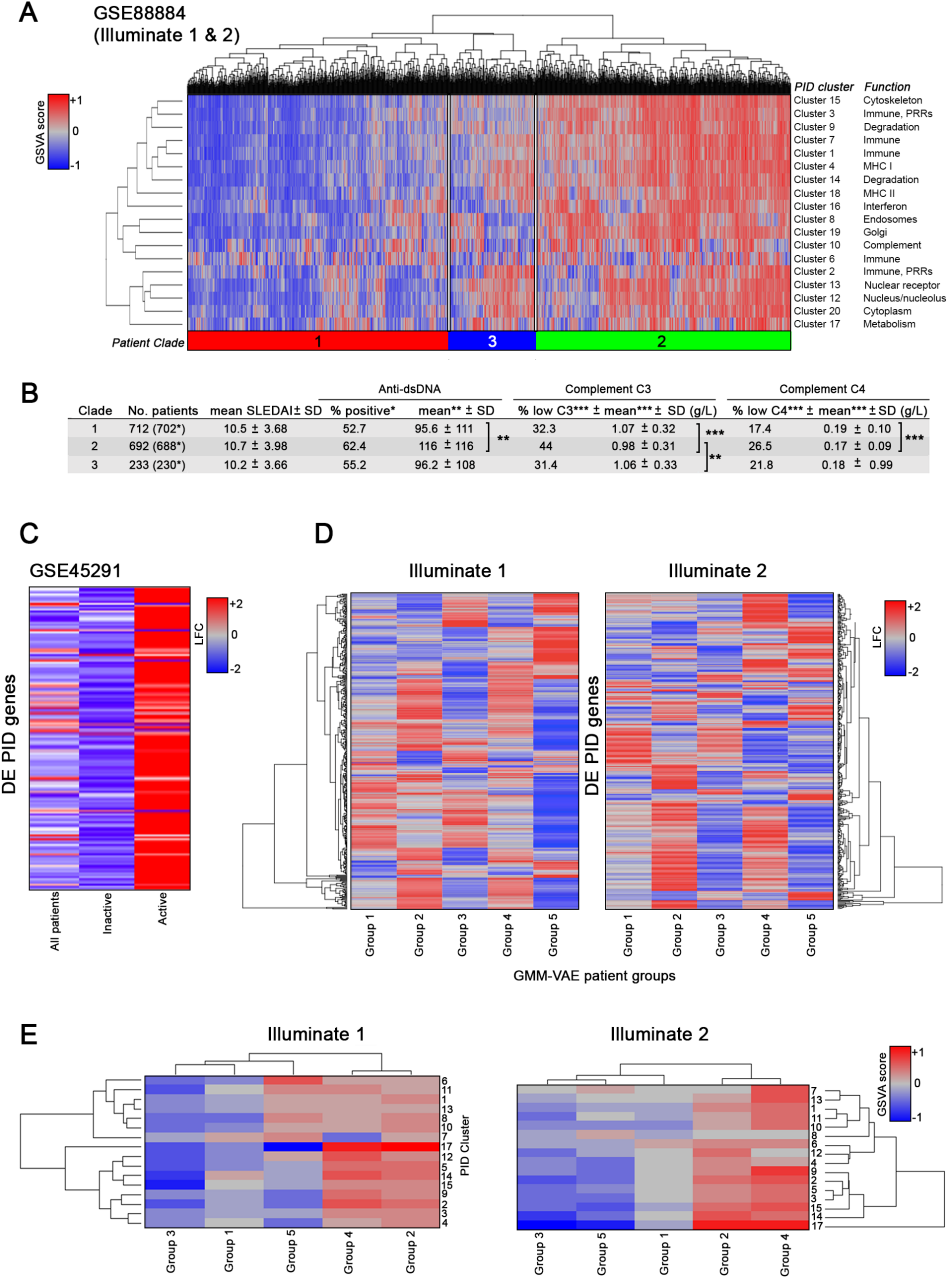
PID mCODE clusters can identify clinically meaningful patient groups. GSVA of SLE patient DE gene data **(A)** using PID mCODE clusters as input gene sets. Output is shown following directed hierarchical clustering set to k=3 (clustering groups are shown as colored and numbered bars between heatmap and dendrogram) after unsupervised hierarchical clustering. **(B)** Clinical data summary and statistics of the three groups resulting from directed hierarchical clustering. Number of patients in parentheses refers to the number of subjects with SLE, versus total number with controls (excluded from analyses). **(C)** Total PID gene DE profile of patients within GSE88884, shown as log fold change, from analysis for all patients combined, inactive (SLEDAI < 6) patients only, or active (SLEDAI ≥ 6) patients only. **(D)** DE values (row z-scores) of PID genes in patient subsets defined by a previous use of a variational autoencoder, separated into Illuminate-1 and Illuminate-2 arms of trial data. **(E)** GSVA enrichment of PID mCODE clusters (row z-score) for each of the five autoencoder-derived groups per trial arm. *p < 0.05; **p < 0.001; ***p < 0.0001.

To explore the enrichment of PID genes in lupus patients in greater detail, we interrogated GSE88884, (Illuminate 1 and 2), a whole blood dataset containing gene expression data from 1,620 active, female SLE patients and 17 controls. The PID gene clusters identified in **Figure 1C** were used to create a collection of 18 gene modules representing immune-related, metabolic and other molecular pathways implicated in primary immunodeficiency. GSVA was applied to determine the relative enrichment of each PID-derived gene signature in GSE88884. Hierarchical sorting of enrichment values produced three major clades of SLE patients, one with generally high module enrichment, a second with modest enrichment and a third with generally low module enrichment (**Figure 6A**).

We hypothesized that the distinct PID expression patterns observed might be related to differences in SLE disease activity. To test this, we analyzed clinical metadata and found patients grouped into the cohort with high mCODE gene module enrichment (clade 2) displayed significantly more active disease as indicated by increased anti-dsDNA titers, decreased circulating C3, and decreased circulating C4 (**Figure 6B**). Consistent with this, clade 2 exhibited high levels of enrichment in the immune-related clusters 1, 3, 7 and 16.

To validate these findings, similar results were obtained from a second dataset (GSE45291) when patients were grouped based upon SLE disease activity index (SLEDAI) and then active and inactive patients examined for differentially expressed PID genes (**Figure 6C**). Finally, as an orthogonal method to validate the relationship between SLE disease activity and enrichment of PID gene modules, we grouped patients from GSE88884 based on clinical features using a GMM-VAE to identify five phenotypic subsets of patients. As shown in **Figure 6D**, differential expression of PID genes was highest in the most active patient groups (2 and 4, **Supplementary Figure S2**) compared with the other groups. Similarly, as shown in **Figure 6E**, enrichment of PID gene modules determined by GSVA was greatest in Group 2, which exhibited the highest mean SLEDAI (12, data not shown), the highest incidence of anti-dsDNA (98%), and the highest incidence of low complement (94%), and Group 4 (**Supplementary Figure S2**). In contrast, Group 3, which contained the least active patients, showed no PID module enrichment, whereas the other patient clusters showed intermediate PID gene module enrichment (**Figure 6E**).

### PID gene modules accurately classify SLE patient disease status

**Figure 7:**
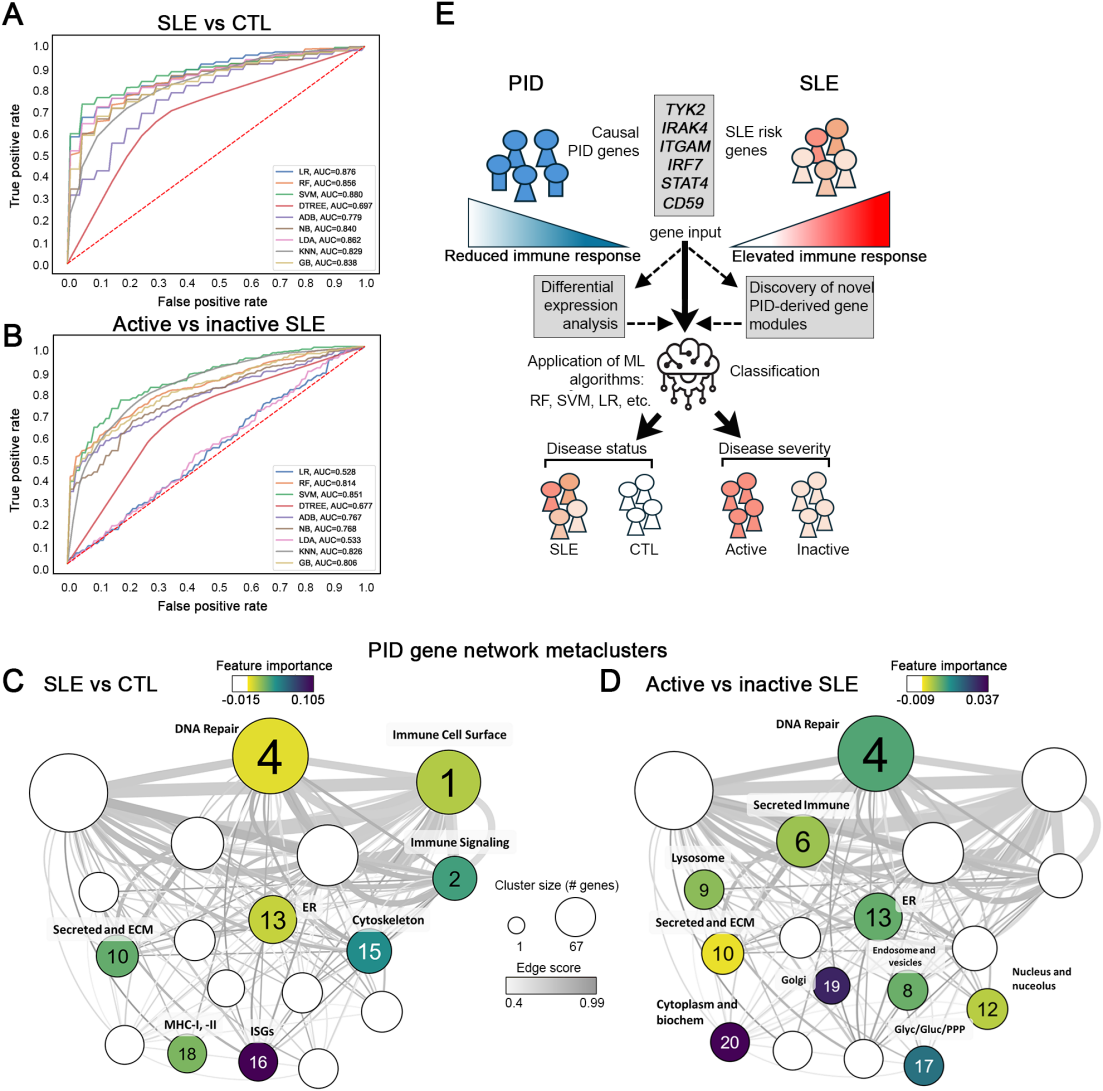
PID gene clusters show utility as ML classifiers for SLE patient disease status. **(A, B)** ROC curves (left) and individual classifier performance statistics (right) for 9 ML classifiers trained using PID mCODE clusters to correctly sort SLE patients from healthy controls **(A)** or active SLE patients from inactive SLE patients **(B)**. **(C)** Top feature clusters for ML identification of SLE vs control or active SLE vs inactive SLE **(D)** across all classifiers. Overall feature importance data is mapped onto the PID mCODE schematic by node color, and clusters with positive feature importance values are annotated by defining BIG-C functional category. **(E)** Schematic illustration showing how PID-derived gene signatures can be used to accurately predict the incidence and severity of SLE, highlighting the opposing nature of the inflammatory pathways essential for host defense and that underlie the development of autoimmune disease.

To determine whether enrichment of PID gene expression could be used to classify patients with SLE, combined GSVA scores for enrichment of each of the 18 PID PPI clusters were calculated for five WB datasets and used to train nine ML classifiers (**Figure 7**). Each ML algorithm attempted to classify subjects based on SLE or control (**Figure 7A**) or active or inactive SLE (**Figure 7B**, **Supplementary Figure S3**) as labels. The ML algorithms effectively classified SLE patients, with the support vector machine (SVM) algorithm achieving the highest accuracy (0.7995) in classifying the SLE cohort from healthy controls (**Figure 7A**; **Supplementary Table S6**) and random forest (RF) being the most effective at classifying active versus inactive SLE patients (accuracy of 0.741) (**Figure 7B**, **Supplementary Table S6**). Notably, feature importance calculation for each comparison revealed that different combinations of mCODE clusters were the most important for accurate classification in each specific comparison; whereas clusters 16 (ISGs), 15 (cytoskeleton), 18 (MHC-I, -II), 2 (immune signaling) and 10 (secreted and ECM) had the highest feature importance in the SLE vs healthy control comparison (**Figure 7C**), prediction of active versus inactive SLE employed clusters 20 (cytoplasm and biochemistry), 19 (Golgi), 4 (DNA repair), and 17 (glycolysis/glucogenesis/pentose phosphate pathway) as the most important (**Figure 7D**). Taken together, these results demonstrate how distinct combinations of PID gene clusters can be used to distinguish between SLE and normal samples and active versus inactive lupus, respectively (**Figure 7E**).

## Discussion

The relationship between PID diseases and autoimmune diseases has long been a subject of interest, discussion, and debate in both fields (23). Many reviews on the subject point out the paradoxical appearance of both PID and autoimmunity within the same patients, and have hypothesized that these conditions share pathological mechanisms and, therefore, represent opposite sides of the same coin (15, 24). However, most of the investigation into these relationships with specific regard to SLE focus on the presentation and treatment of PID concomitant with SLE, not with mechanisms of SLE pathogenesis that are driven by PID gene dysregulation (18, 25).

We employed a database of 453 causal PID genes to interrogate patients with SLE and found that these immune response checkpoint genes are disproportionately represented among genes known to increase risk of SLE and among genes that are significantly differentially expressed or enriched in SLE. Although some PID conditions have been associated with features of autoimmunity, these results firmly establish that the family of genes whose loss of function is non-redundantly causative of increased susceptibility to infectious agents positively contribute to SLE pathogenesis. Many of these genes overlap with SISRGs. Similarly, PID genes overlap with both RA- and MS-associated genes, but the degree of overlap with SLE-associated genes is substantially greater. A majority are over-expressed in SLE compared to healthy donors and even more over-expressed in active SLE. Finally, the association of PID genes with SLE pathogenesis is sufficiently robust that they can be used to classify SLE from healthy controls or active from inactive SLE by ML. Together, these data strongly indicate that the genes identified as being causative of altered host defense to microbial pathogens are primarily involved in the enhanced immune responsiveness underlying SLE.

It was notable that there was a significant overlap between PID genes and risk genes for SLE, but less so for RA- or MS- associated genes. eQTL analysis combined with predictions from intergenic enhancer mutations, coding-region variants, and SNP-gene proximity orthogonally identified a group of predicted SLE risk genes (4, 5), and a disproportionate percentage of the PID genes were found to overlap with these SISRGs. Within this overlap, several genes belonged to pathways and processes known to contribute to lupus pathogenesis, such as apoptosis, interferon and other cytokine signaling, the complement system, as well as anti-inflammation (**Supplementary Figure S4**). Gene products involved in signal transduction, DNA repair, metabolism, regulation ofimmune processes and the formation of protein structures, were also identified among the overlapping genes and may constitute new targets of therapy (**Supplementary Table S5**). These findings indicate that genes identified as providing a non-redundant risk for susceptibility to microbial pathogens contribute to the likelihood of developing SLE and include a number of targets of approved drugs for other indications or drugs in development (**Supplementary Figure S4**). Although the contribution of each of these genes to SLE pathogenesis is small in contrast to their capacity to cause a specific defect in host defense, the results clearly establish a role for these genes in the genetic underpinning of lupus immunopathogenesis.

It is also notable that PID genes are also more likely to be differentially expressed in peripheral blood of SLE patients when compared to random gene cohorts, and this highly significant enrichment holds at multiple levels of filter stringency and across multiple thresholds of Monte Carlo simulation repetition, corroborating the hypothesis that PID genes are clearly over-expressed in SLE. The cellular distribution of PID gene-altered expression observed in SLE echoes findings of previous studies that examined the link between SLE and perturbations in specific immune cell populations, including T cells and myeloid cells (18, 26). Particularly noteworthy are significant shifts in expression patterns of PID genes associated with classical and nonclassical monocyte subpopulations that have been implicated as drivers of SLE activity and flares (27, 28). Moreover, tissue inflammation in SLE is characterized by the involvement of distinct immune cell subtypes, including monocytes and myeloid cells (22, 29–32). Further study will be required to causally link dysregulation of PID genes in these specific immune cell types with organ system involvement, disease activity, and patient endotype group membership.

Furthermore, SLE-specific expression of PID genes is related to disease activity although enrichment of PID gene expression is also observed in SLE patients with low disease activity. Notably, enrichment of the expression of PID genes in both active and inactive lupus is sufficiently robust to serve as the ML features to classify both SLE from normal and active SLE from inactive disease. The specific patterns of gene expression, however, that serve as the strongest features to discern SLE from control differ from those that classify active versus inactive SLE. PID genes in immune signaling pathways, MHC-related pathways, cytoskeleton pathways, and especially the interferon gene signature are uniquely identified as top factors that identify SLE from control. In contrast, mCODE clusters enriched in lysosomal, endosomal, metabolic, Golgi-related, and cytoplasm/biochemistry-related PID genes are uniquely defining features between active and inactive SLE. Many of these functional clusters contain individual genes that have been previously linked to SLE disease activity (19, 22, 33), but in this work we uniquely outline a method of ML-driven disease state detection using gene clusters exclusively comprised of PID genes, maximizing the immunological information content of the ML input and, therefore, its utility. These findings demonstrate that although PID genes are broadly over-expressed in SLE, the nuanced expression patterns they display can be used to track disease progression and activity.

As shown in this work, generation and application of functional gene clusters not only accomplishes the dimensionality reduction required to shrink a large signature into a sufficiently small enough number of deterministically significant genes to be reasonable for diagnostic test development, but also can also identify subgroups of patients that do not appear in bulk differential expression analysis and can better separate patients on the basis of actual clinical features. Whereas a bulk of disease impact is related to the disease activity of the patient, SLEDAI scoring may in fact obscure differences in clinical features/symptoms or exacerbation (34, 35). We tested this hypothesis by applying a variational autoencoder to the 1620 patients from the GSE88884 dataset to evaluate the degree to which disease activity (and the clinical variables from which it is derived) was related to expression of PID genes. The autoencoder sorted SLE patients into five groups based on the presence and/or absence of defined clinical parameters. Notably, PID gene expression appeared to also track with these groups: 89% of PID genes were significantly differentially expressed among the five patient groups, with over-expression of genes in more active patients, decreased expression in somewhat inactive patients, and variable expression in one group potentially related to the presence of lymphopenia. Enrichment of the mCODE gene modules within these patient groups showed similar distributions, i.e., active patient groups assemble together and inactive patient groups assemble together when subjected to hierarchical clustering. This finding reinforces the conclusion that specific, unique combinations of PID genes are directly related to patient outcomes and disease severity.

### Clinical significance

SLE is a polygenic disease with each non-MHC risk allele contributing a small increase in the chance of developing SLE. It is widely acknowledged that serological or laboratory-based biomarkers for lupus are suboptimal for identifying immunologic activity (36). For example, ANA tests have a high overall sensitivity (94%) but relatively low specificity (61%), thereby increasing the likelihood of false positive diagnoses. On the other hand, assessment of anti-dsDNA and anti-Sm antibodies provides good specificity but low sensitivity. In contrast, ‘omics-based biomarker discovery has the advantage of testing many different combinations of genes and gene signatures capable of assessing and/or monitoring disease status. It is, therefore, notable that the PID genes convey sufficient risk of developing SLE that they can be used as features in ML to classify the disease from normal and also active from inactive SLE. Although it is known that the confluence of SLE risk alleles can increase the likelihood of developing SLE (37), the contribution of PID genes to SLE risk seems out of proportion to that contributed by random genes or even an aggregate of SLE risk alleles. This is consistent with the conclusion that PID genes encode a unique set of immune checkpoint molecules that disproportionately contribute to SLE risk. Not only does this emphasize the immune nature of SLE risk, but it also identifies target pathways that could be employed as novel ways to intervene in this autoimmune disease. By way of example, DNA repair arose as a prominent feature in both the ML classifier constructed to distinguish SLE from non-SLE patients and active SLE from inactive SLE patients. This may suggest that therapeutics targeting regulators of DNA repair mechanisms would be a good focus for future study in treating active SLE. Once a patient is categorized as a member of a specific PID gene module-informed subgroup, agents selectively targeting gene perturbations might be considered (38–40).

## Limitations and conclusions

Limitations of this study include those related to the data integrated in our analyses. SLE genetic association studies have been restricted in size and scope and are heavily biased toward European ancestry populations. Nonetheless, to maximize both power and scope, we used the largest genetic association study for SLE, which is limited to Immunochip SNPs (37) and the largest SLE GWAS (4, 5). For gene expression analyses, datasets were selected based on the size of their patient population, the presence of healthy control samples in addition to SLE patient samples, and the relevant purified cell type or tissue of origin of the samples. However, the majority of these datasets do not contain additional clinical information preventing analyses of PID enrichment with specific SLE phenotypes and/or comorbidities, such as lupus nephritis or cardiovascular disease. We would point out that although this study is based on bioinformatics and computational approaches, many of the findings were consistent across multiple datasets, with differentially expressed PID gene-based signatures able to separate SLE from control subjects in 2 different whole blood datasets. It would, however, be useful to test some of the lupus associated PID-derived genes in animal models to confirm their role in disease pathogenesis. Taken together, these results show that PID gene-derived signatures can be used to identify incidence and activity of SLE with very high accuracy. Quantifying PID gene expression and PID gene cluster enrichment may therefore be the basis of focused and directed testing to stratify SLE patients and track disease progression and severity with detail that surpasses current methodology.

## Data Availability

The original contributions presented in the study are publicly available. This data can be found here: https://figshare.com/s/e550eaac8fcdd1d12bc4.

## Supplementary material

Supplementary Figure 1 BIG-C enrichment of PID PPI network. BIG-C was used to annotate functional PID clusters generated from PPI network construction. Genes comprising each of the 20 PID clusters were input into a custom typescript application and enrichment statistics for BIG-C categories were calculated. Only significant results by Fisher’s Exact test are plotted as shown. Odds ratio is shown by bubble size and significance is shown by bubble color shading as -log(p).

Supplementary Figure 2 Clinical characteristics of GMM-VAE patient groups. Percentage of patients systemic traits of lupus by patient group defined by a variational autoencoder.

Supplementary Figure 3 ML performance of classifiers of active or inactive SLE. ROC curves and AUC values for nine classification algorithms using PID gene enrichment as input. Five whole blood datasets underwent GSVA using 18 PID PPI clusters, and enrichment scores were concatenated. A total of 1665 active and 242 inactive patient data were used in training and test datasets. Subsampling was implemented to handle the data imbalance, with random shuffling and selection of 242 active SLE samples matched with inactive lupus samples. 7 subsets or folds were obtained and the mean ROC of these folds were plotted in addition to performance measures of individual folds.

Supplementary Figure 4 Characterization of PID gene overlap with SNP-implicated SLE risk genes. Summary of known pathogenic pathways and processes and novel functions represented by the gene products in the overlap of PID genes with SNP-implicated SLE risk genes. The list of 129 overlapping genes was also queried for targets of current drugs in development or drugs with FDA approval.
