# Supplementary figures and images for: Genes causative of primary immunodeficiency are risk factors for and are over-expressed in systemic lupus erythematosus
Source: Front Immunol. 2026 Mar 3;17:1494343. doi: 10.3389/fimmu.2026.1494343 (PMC13040371; doi:10.3389/fimmu.2026.1494343)

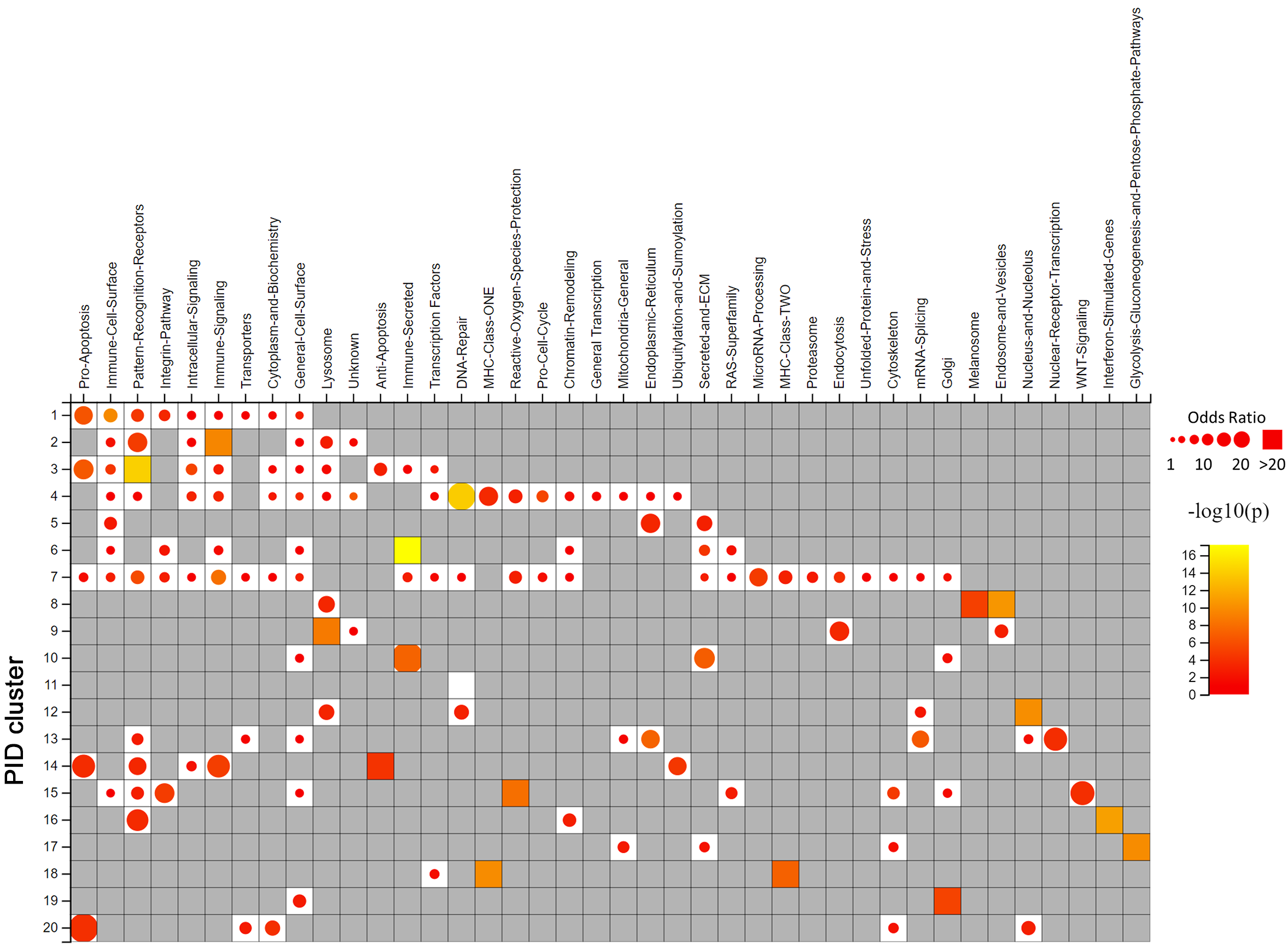

Supplement: Supplementary Figure 1 — BIG-C enrichment of PID PPI network. BIG-C was used to annotate functional PID clusters generated from PPI network construction. Genes comprising each of the 20 PID clusters were input into a custom typescript application and enrichment statistics for BIG-C categories were calculated. Only significant results by Fisher’s Exact test are plotted as shown. Odds ratio is shown by bubble size and significance is shown by bubble color shading as -log(p). [file Image1.tif]

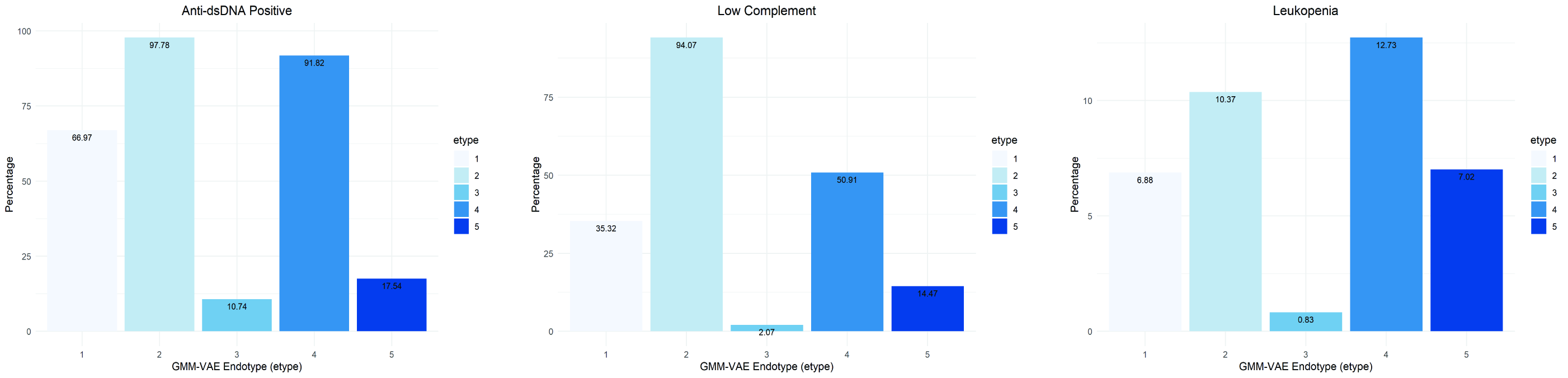

Supplement: Supplementary Figure 2 — Clinical characteristics of GMM-VAE patient groups. Percentage of patients systemic traits of lupus by patient group defined by a variational autoencoder. [file Image2.tif]

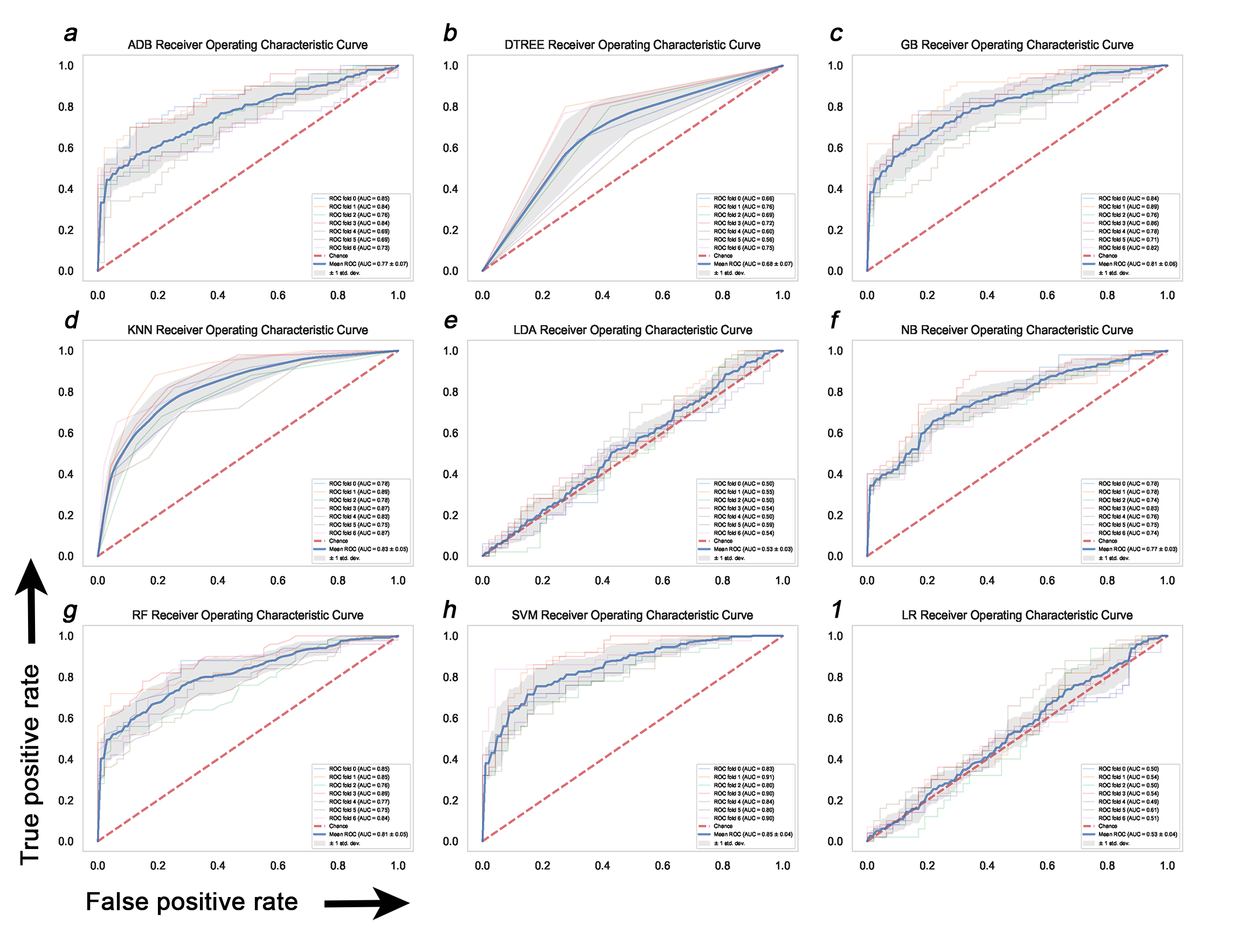

Supplement: Supplementary Figure 3 — ML performance of classifiers of active or inactive SLE. ROC curves and AUC values for nine classification algorithms using PID gene enrichment as input. Five whole blood datasets underwent GSVA using 18 PID PPI clusters, and enrichment scores were concatenated. A total of 1665 active and 242 inactive patient data were used in training and test datasets. Subsampling was implemented to handle the data imbalance, with random shuffling and selection of 242 active SLE samples matched with inactive lupus samples. 7 subsets or folds were obtained and the mean ROC of these folds were plotted in addition to performance measures of individual folds. [file Image3.tif]

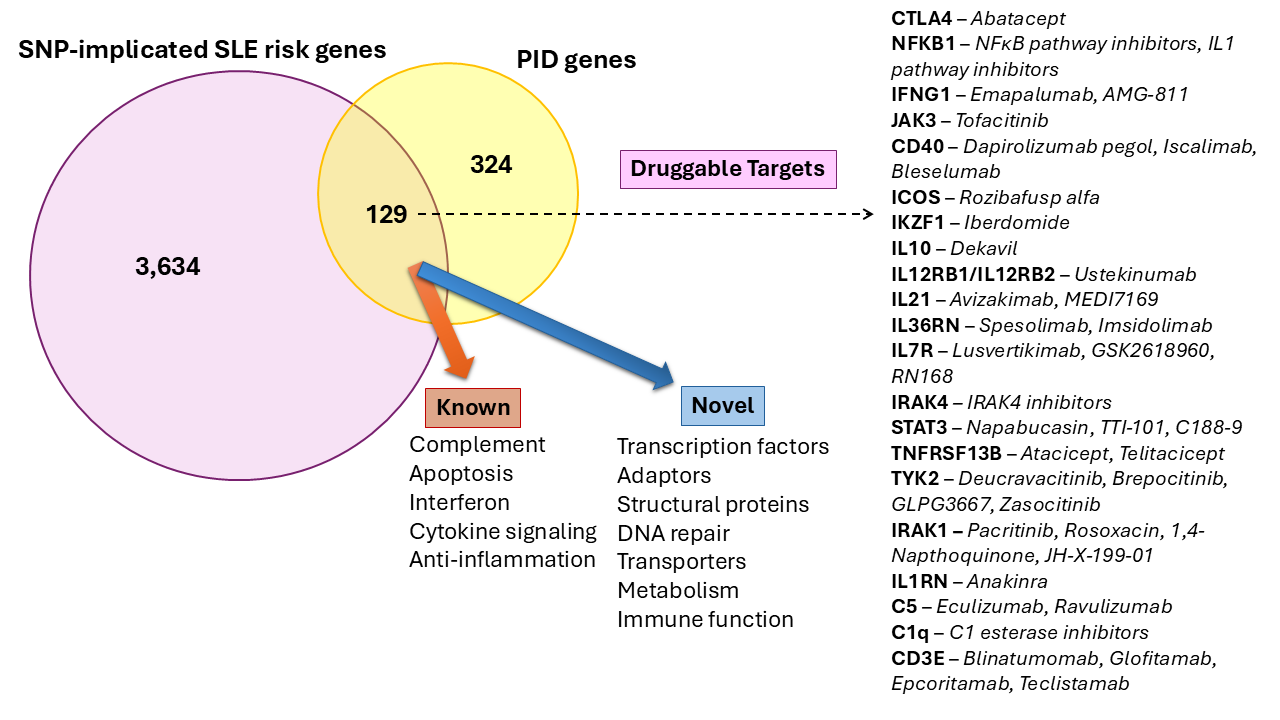

Supplement: Supplementary Figure 4 — Characterization of PID gene overlap with SNP-implicated SLE risk genes. Summary of known pathogenic pathways and processes and novel functions represented by the gene products in the overlap of PID genes with SNP-implicated SLE risk genes. The list of 129 overlapping genes was also queried for targets of current drugs in development or drugs with FDA approval. [file Image4.tif]
